# A robust method for measuring an individual’s sensitivity to facial expressions

**DOI:** 10.3758/s13414-020-02043-w

**Published:** 2020-05-08

**Authors:** Louise S. Delicato

**Affiliations:** 1 Department of Psychology, School of Social Sciences, Heriot-Watt University, Edinburgh, EH14 4AS UK; 2 School of Psychology, University of Sunderland, Sunderland, UK

**Keywords:** Emotion recognition, Faces, Individual, Discrimination, Duration, Image size, Morph, *n*-of-1

## Abstract

This paper describes a method to measure the sensitivity of an individual to different facial expressions. It shows that individual participants are more sensitive to happy than to fearful expressions and that the differences are statistically significant using the model-comparison approach. Sensitivity is measured by asking participants to discriminate between an emotional facial expression and a neutral expression of the same face. The expression was diluted to different degrees by combining it in different proportions with the neutral expression using morphing software. Sensitivity is defined as measurement of the proportion of neutral expression in a stimulus required for participants to discriminate the emotional expression on 75% of presentations. Individuals could reliably discriminate happy expressions diluted with a greater proportion of the neutral expression compared with that required for discrimination of fearful expressions. This tells us that individual participants are more sensitive to happy compared with fearful expressions. Sensitivity is equivalent when measured on two different testing sessions, and greater sensitivity to happy expressions is maintained with short stimulus durations and stimuli generated using different morphing software. Increased sensitivity to happy compared with fear expressions was affected at smaller image sizes for some participants. Application of the approach for use with clinical populations, as well as understanding the relative contribution of *perceptual processing* and *affective processing* in facial expression recognition, is discussed.

This paper describes a method to measure the sensitivity of an individual to different facial expressions and test differences in the individual’s sensitivity to these facial expressions using the model-comparison approach. This method can be applied to both (i) clinical areas where case studies are important and access to large sample sizes can be challenging and (ii) understanding the relative contribution of *perceptual processing* and *affective processing* in facial expression recognition. Where perceptual processing is mainly driven by sensory systems and relies on the visual information in the face, affective processing retrieves emotional meaning through higher-order cognitive processes.

Facial expressions are widely thought of as external representations of an individual’s internal thoughts, motivations, and feelings. Therefore, reliable detection and identification of the emotion conveyed by facial expressions is essential for effective positive social communication. Additionally, there is considerable evidence showing that ability to recognize emotions from facial expressions is altered or impaired in, among others, people with depression (Dalili, Penton-Voak, Harmer, & Munafò, 2015), autism spectrum disorders (Trevisan & Birmingham, 2016), and neurodegenerative disorders (Löffler, Radke, Morawetz, & Derntl, 2016). Therefore, understanding how sensitive an individual is to different facial expressions could have diagnostic value and/or support the monitoring of treatment regimen.

Facial emotion recognition relies on both perceptual processing and processing of affect from nonsensory systems, the relative contribution of which remains unclear (see Calvo & Nummenmaa, 2016, for a review). Dissociating the effect of perceptual processing from affective processing in facial expression recognition might be achieved by using stimuli that are tailored to individual participants. Measures of sensitivity to different expressions could be used to identify stimuli from different affective categories (e.g., happy and fear) that individual participants find equally challenging to discriminate (perceptually equivalent). Such perceptually equivalent stimuli could then be used in another experimental paradigm to measure the effect of *affect* on performance. Tailoring the stimuli to individual participants would influence the relative contribution of perceptual and affective processing in the paradigm. Using perceptually equivalent stimuli in a different paradigm (e.g., categorial emotion recognition task) would reduce the contribution of the salience of the visual signals (perceptual processing) and amplify the relative contribution of affective processing. Comparing performance with and without perceptually equivalent stimuli could provide greater insight into the relative contribution of perceptual and affective processing in facial emotion recognition tasks. This paper presents a way to measure the sensitivity of an individual to different facial expressions and therefore identify perceptually equivalent stimuli tailored to individuals.

Emotion recognition from facial expressions has been studied extensively using a wide range of methodological approaches. Much of this research has focused on categorization of expressions where participants are asked to identify the emotion conveyed by one of (usually) six *universal* expressions (happiness, anger, disgust, fear, sadness, and surprise), often at full intensity. Results consistently show that performance in a categorical task was more accurate and faster for happy than for fearful expressions, while performance with the other universal expressions (anger, disgust, sadness, and surprise) was usually in between (Calder, Keane, Young, & Dean, 2000a; Calvo & Lundqvist, 2008; Calvo & Nummenmaa, 2009; Matsumoto & Hwang, 2014; Palermo & Coltheart, 2004; Recio, Schacht, & Sommer, 2013).

In addition to studies using faces with fully formed expressions (full intensity), morphing techniques have been used to generate face stimuli with varying intensities of expression. Computer software is used to morph between a neutral face and a face of the same individual with a full expression. Images at intermediary stages are saved creating new images of expressions with intermediate intensities. Such stimuli are arguably more ecologically valid as emotions expressed in everyday life vary in their intensity. Positive correlations between degree of morphing and intensity rating (Calder, Rowland, et al., 2000b) and accuracy recognizing emotions (Hess, Blairy, & Kleck, 1997) have been found for static faces. Dynamic morphed stimuli have also been used to identify the intensity at which an expression is detectable or able to be categorized (Fiorentini, Schmidt, & Viviani, 2012; Fiorentini & Viviani, 2011; Niedenthal, Brauer, Halberstadt, & Innes-Ker, 2001; Niedenthal, Halberstadt, Margolin, & Innes-Ker, 2000).

Participants find it easy to discriminate facial expressions irrespective of whether they are asked to categorize or discriminate between stimuli with different emotional content (Calvo, Fernández-Martín, & Nummenmaa, 2012) or whether stimuli with the same or variable emotional intensities are used (Calder, Rowland, et al., 2000b; Calvo, Avero, Fernández-Martín, & Recio, 2016; Hess et al., 1997; Marneweck, Loftus, & Hammond, 2013). Performance on a recognition task is good (above chance) even when stimuli are presented at very short durations (<50 ms; Calvo & Lundqvist 2008), or when key features of the face are disrupted (Bombari et al., 2013; Calder, Rowland, et al., 2000b; Calvo, Fernández-Martín, & Nummenmaa, 2014; Delicato & Mason, 2015; Tanaka, Kaiser, Butler, & Le Grand, 2012).

However, while people are generally good at discriminating expressions, sensitivity to morphed faces is affected by, among others, psychopathy, social anxiety, nonsuicidal self-harm, schizophrenia, and whether an individual exhibits manic or depressed symptoms of bipolar disorder (Blair, Colledge, Murray, & Mitchell, 2001; Blair et al., 2004; Gray et al., 2006; Heuer, Lange, Isaac, Rinck, & Becker, 2010; Shah et al., 2018; Ziebell, Collin, Weippert, & Sokolov, 2017). Therefore, developing an approach that can reliably measure an *individual’s* sensitivity to different facial expressions could have value in a clinical context where case studies are important and where it can be difficult to recruit large samples.

Marneweck, Palermo, and Hammond (2014) showed that a psychophysical approach can be used to measure sensitivity to different emotions for groups of participants using morphed stimuli (for a similar approach, see Calvo et al., 2016). The authors presented pairs of faces on a computer screen, one after the other, and asked participants to discriminate between them. One of the faces was neutral, whereas the expression of the other face varied between neutral (0%) and 35% expressive. Performance on the task increased as the intensity of the expression increased for each emotion (happy, anger, disgust, and sad). A psychometric function, a curve describing the relationship between performance and stimulus intensity, was fit to individual participants’ data, and estimates of threshold and slope were obtained. Group analysis showed that people with Parkinson’s found it more difficult to discriminate between neutral and expressive faces compared with controls.

A common theme across research in facial expression recognition is that behavioural performance is reported as a mean of a group of participants and analyzed with sample means and statistics at a group level (often using least-squares methods; e.g., *t* test, ANOVA). Marneweck et al. (2013; Marneweck et al., 2014) showed that a psychophysical approach can also be used to investigate expression recognition in this way. However, analysis at a group level does not utilize the full potential of the psychophysical approach.

Psychophysical methodology is the foundation of our understanding of the mechanisms of sensation and perception. It allows precise measures of performance to be made using multiple trials with different, but equivalent, stimuli *within a single individual*. There are two methodological advantages of this approach: (i) It is sensitive enough to measure differences that the participant is not aware of and (ii) data can be analyzed at the level of the individual. This paper describes a method to measure the sensitivity of *an individual* to different facial expressions as well as a method to test whether these differences are statistically significant for the individual.

The approach described by Marneweck et al. (2013) can be developed to reliably measure an *individual’s* sensitivity to different facial expressions. In their study, the intensities of the stimuli and the steps between intensities were held constant across all participants. This meant that it was not always possible to fit a psychometric function to each individual’s data, and therefore some participants data could not be included in the group analysis. In this study, stimuli will be presented with finer gradations and with intensities that are tailored to the individual to optimize the possibility for a function to be fit to each individual’s data.

This paper goes further to show how the model-comparison approach (Prins & Kingdom, 2018) can be used to test for *significant differences in the sensitivity of the individual* to two different facial expressions (happy and fear). The model-comparison approach tests the hypothesis that differences between the psychometric functions fit to an individual’s performance with happy and fear expressions are *real* rather than due to sampling error. It establishes whether the underlying data are distinct and belong to two models (alternative hypothesis)—Model 1: neutral versus happy, and Model 2: neutral versus fear, or, whether the data are identical (null hypothesis) and belong to a single model: neutral versus (any) expression. This approach to analysis has particular value for use with case studies and/or clinical contexts in which it may be difficult to recruit the sample size needed to achieve statistical power using the least-squares approach (e.g., *t* test, ANOVA) typically associated with psychology studies. It may be of particular value to the growing number of *n*-of-1 studies whose aim is to support the development of personalized health behaviour interventions (see McDonald et al., 2017, for a review).

The effect of stimulus duration and image size on an individual’s sensitivity to facial expressions is also evaluated. It is important to understand the conditions in which the effect is observed in order to evaluate the potential of the application of the approach outside of the lab (e.g., online) and/or in a clinical context where control of some parameters is more difficult. Finally, the paper explores how software, and the process used to generate stimuli, may also have an impact on sensitivity to the expressions. Preliminary data have been presented in abstract form (Delicato, Finn, Morris, & Smith, 2014).

## Method

### Participants

A total of 20 participants were recruited through volunteer sampling from the University of Sunderland undergraduate population. Eleven participants took part in Experiment 1, which investigated sensitivity to happy and fear analyzed at the group and individual level. A subset of these 11 participants (*n* = 6) took part in Experiment 2, which investigated whether sensitivity to happy and fear depended upon unique faces. A further subset of this group (*n* = 3) took part in Experiment 3, which investigated the effect of stimulus duration, and Experiment 4, which investigated the effect of image size on sensitivity to happy and fear. Nine different participants took part in Experiment 5, which investigated the effect of the morphing software used to generate stimuli on sensitivity to happy and fear. All participants had normal or corrected-to-normal vision. The research was conducted in accordance with The Code of Ethics of the World Medical Association (Declaration of Helsinki) and received approval from the University of Sunderland Research Ethics Committee.

### Stimuli

**Fig. 1:**
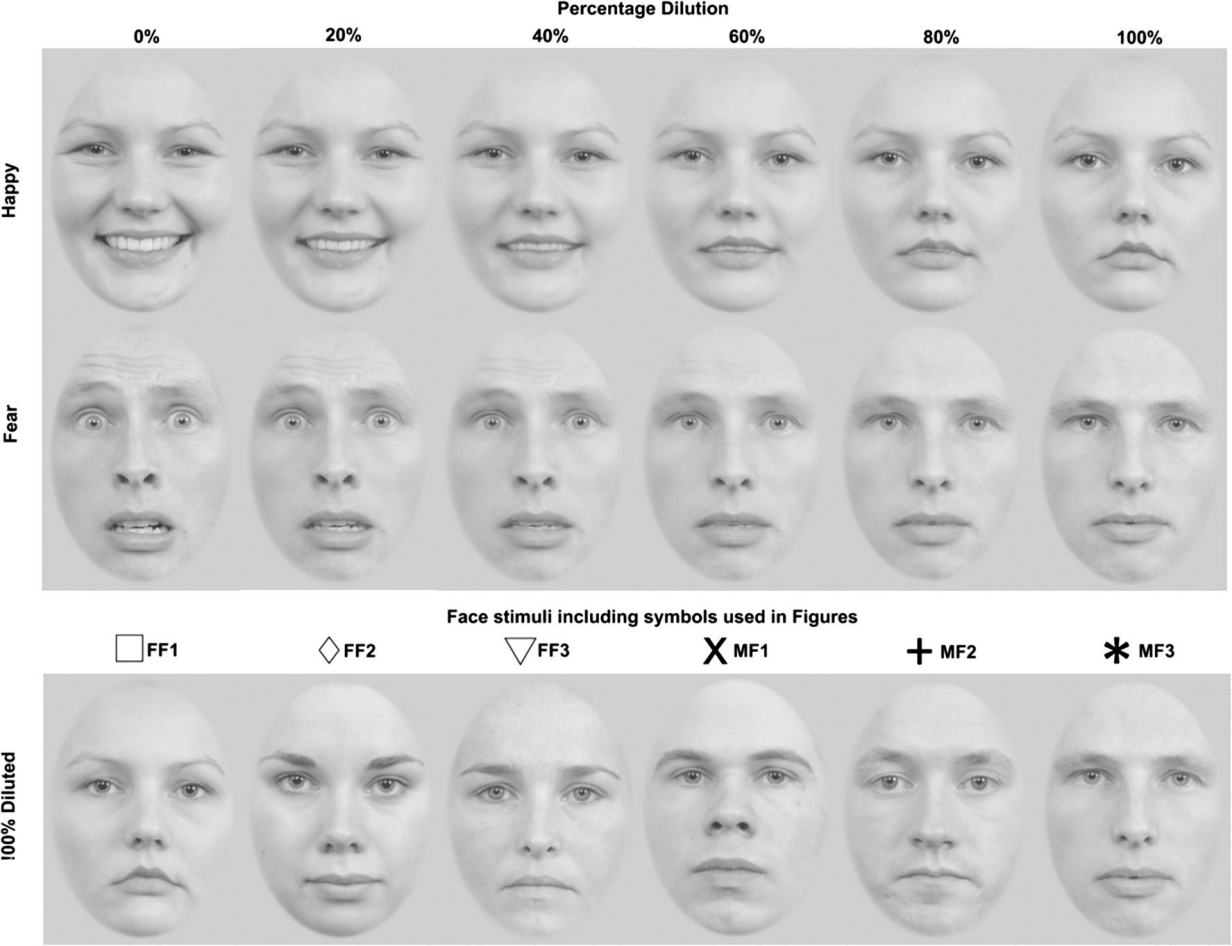
Examples of happy (top row) and fearful images (middle) used as stimuli for a range of stimulus dilutions (0%–100%). Stimulus dilution increases from left to right showing how the full intensity expression (0%) changes with increasing dilution until the image becomes neutral (100%). The bottom row provides examples of the neutral images (100%) of each face with corresponding symbols used to plot data in Figs. 5b and 6. There are three female faces (open symbols; FF1, FF2, and FF3) and three male faces (symbols; MF1, MF2, and MF3)

Stimuli were six unique White Dutch faces, three males and three females (RaFD 09, 10, 71, 08, 19, and 61) obtained from the Radboud Face Database (RaFD; see Fig. 1; Langner et al., 2010). All unique faces express two basic emotions, happy and fear, as well as neutral, captured from a frontal viewpoint with eyes directed towards the camera under supervision from a Facial Action Coding System specialist. The mean intensity ratings averaged across all faces used are identical for happy (4.25) and fearful expressions (4.25; see Supplementary Material in Langner et al., 2010).

#### Experiments 1–4: Stimuli generated using Norrkross MorphX

Original images were converted to greyscale, masked with an oval shape (620 × 880 pixels) to remove any external features (e.g., hair, ears) using Adobe Photoshop CS5. Photoshop was used to adjust the brightness of each unique face so that the mean intensity value in the histogram of the image in Photoshop was the same for each unique face.

Stimulus dilutions ranging between 2% and 98% in steps of 2% were generated by morphing between the original full expression (0% diluted) and neutral (100% diluted) image corresponding to each unique face (RaFD 09, 10, 71, 08, 19, and 61) using Norrkross MorphX (Marneweck et al., 2013; Wennerberg, 1997). A minimum of 29 corresponding feature locations on the neutral and full expression images were used to generate the stimulus dilutions. All stimuli were presented on a uniform mid-grey background.

For Experiments 1 and 2, stimuli were presented on screen for 200 ms at a viewing distance of 50 cm generating an image size of 19° × 27° of visual angle. For Experiment 3, stimuli were presented on screen for 8 ms, 83 ms, or 200 ms at a viewing distance of 50 cm, generating an image size of 19° × 27° of visual angle. For Experiment 4, stimuli were presented for 200 ms at a viewing distance of 50, 200, or 400 cm. These viewing distances generated images that subtended 19° × 27°, 5° × 7°, and 2.5° × 3.5° of visual angle, respectively.

Following Marneweck et al. (2013), seven levels of stimulus dilution were varied to characterize the relationship between performance and stimulus dilution for each participant and for each expression (happy and fear). The precise stimulus dilutions presented varied by participant to ensure full characterization of performance, and there were at least 40 observations per stimulus dilution to enable fit of a psychometric function for each expression. For six of the eleven participants in Experiment 1, there were 240 observations per stimulus dilution per participant (40 repeats per face stimulus for six unique face stimuli). This allowed a psychometric function to be fit to each unique face (Experiment 2). There were 240 observations per stimulus dilution investigating the effect of stimulus duration (Experiment 3) and image size on sensitivity to happy and fear (Experiment 4).

#### Experiment 5: Stimuli generated using Psychomorph

Stimulus dilutions ranging between 1% and 99% in steps of 1% were generated by morphing between the original full expression (0% dilution) and neutral (100% dilution) image corresponding to each unique face (RaFD 09, 10, 71, 08, 19, and 61) using Psychomorph (Tiddeman, Burt, & Perrett, 2001). A total of 189 corresponding feature locations on the neutral and full expression images were identified, and stimuli were created using the methods developed by Benson and Perrett (1991). Stimuli were then converted to greyscale, masked with an oval shape (277 × 387 pixels) to remove any external features (e.g., hair, ears) using Adobe Photoshop CS5. Stimuli were matched for mean luminance in MATLAB using the SHINE toolbox (Willenbockel et al., 2010). All stimuli were presented on a uniform mid-grey background.

Stimuli were presented on screen for 200 ms at a viewing distance of 50 cm so that images subtended 17.6° × 24.5° of visual angle. Seven levels of stimulus dilution were varied to measure the relationship between performance and stimulus dilution for each participant and for each expression (happy and fear). Performance is based on at least 40 observations per stimulus dilution per participant.

### Apparatus

Stimuli were controlled using MATLAB 7.7.0 (R2008b) and Psychtoolbox 3.0.8 routines on a Mac Pro Quad Core 2.8 GHz computer with 3 GB RAM and an ATI Radeon HD 5770 graphics card with 1 GB memory. The stimuli were presented on a Samsung Syncmaster 2233RZ monitor with a resolution of 1,680 × 1,050 and a refresh rate of 120 Hz.

### Procedure

**Fig. 2:**
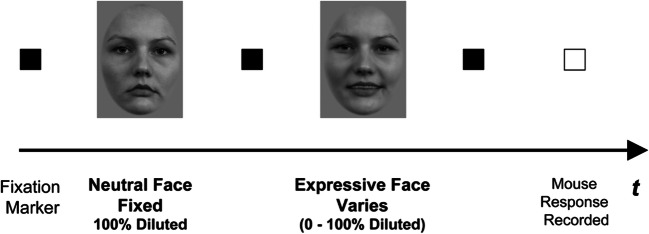
Temporal two-interval forced-choice procedure where the neutral face (comparison) is presented in the first interval and the expressive face (test) in the second interval. Participants were prompted by the question “Which interval contained the face with the greatest expression, the first or second?” and responded with a single click of the mouse for the first interval and a double mouse click for the second

Participants were tested in a dark room lit only by the light of the monitor. Pairs of faces were presented on a computer screen, one after the other, using a temporal two-interval forced-choice procedure and method of constant stimuli (see Fig. 2). On a single trial, two faces, a neutral face (comparison stimulus) and an expressive face (test stimulus), were presented (same unique face; see Fig. 1). Each trial was initiated by pressing a mouse button; the test stimulus was presented in one interval and the comparison in the other. The strength of the test stimulus varied between 0% and 100% dilution, while the comparison was always 100% diluted. The test stimulus had a 0.5 probability of being presented in the first interval. Participants fixated in the centre of the screen and indicated, by pressing the mouse, whether the test stimulus appeared in the first or second interval. Participants were prompted by the question “Which interval contained the face with the greatest expression, the first or second?” The question was identical for each expression (happy and fear), and participants responded with a single click of the mouse for the first interval and a double click for the second. The whole set of stimuli was presented *n* times before any member of the set was presented *n + 1* times.

### Data analysis

#### Fitting psychometric functions

Separate psychometric functions for each individual participant for each experimental condition (e.g., emotion/unique face/stimulus duration/image size) were fit using a Logistic function with the Palamedes toolbox (Prins & Kingdom, 2018) for MATLAB. The Logistic function took the form

1 $$ {F}_L\left(x;\alpha, \beta \right)=\frac{1}{1+\mathit{\exp}\left(-\beta \left(x-\alpha \right)\right)}, $$

where *α* corresponds to the threshold: *F*_*L*_(*x* = *α*; *α*, *β*) = 0.75, and *β* to the slope of the function.

Sensitivity is defined as measurement of the proportion of neutral facial expression in a stimulus required for participants to discriminate the emotional expression on 75% of presentations. The graphical representation of the fitted function is mirrored in the Results section so that an increase in sensitivity is represented by a rightwards shift in the psychometric function, and performance decreases as stimulus dilution increases.

#### Testing for significant differences within the individual

A model comparison using the likelihood ratio test of the Palamedes toolbox (Prins & Kingdom, 2018) for MATLAB was used to test the hypothesis that the differences between the psychometric functions fit to an individual’s performance with happy and fear expressions are real rather than due to sampling error. It establishes whether the underlying data are distinct and belong to two models (alternative hypothesis)—Model 1: neutral versus happy, and Model 2: neutral versus fear, or, whether the data are identical (null hypothesis) and belong to a single model: neutral versus (any) expression.

Palamedes toolbox fits the data from both expressions twice—once as though it belongs to two models (2PF; alternative hypothesis) and once as though it belongs to one model (1PF; null hypothesis). The likelihood ratio is a measure of the fit of each of the models (2PF versus 1PF); the smaller the likelihood ratio, the poorer the fit of 1PF and, therefore, more likely the model is 2PF (alternative hypothesis).

Simulation of an observer is repeated 10,000 times for each expression, allowing the threshold and slope to vary while the guess and lapse rates are fixed. For every repetition the likelihood ratio is calculated based on the simulated results, and it is checked whether the likelihood ratio is as small as those obtained from the individual’s data. The number of simulations out of 10,000 where the likelihood ratio is smaller than that based on the individual’s data is converted into a *p* value. Fewer than 500 simulations out of 10,000 where the likelihood ratio is smaller than that based on the individual’s data would generate a *p* value of *p* < .05 and is a significant difference.

## Results

### Experiment 1: Sensitivity to happy and fearful expressions at the group and individual level

**Fig. 3:**
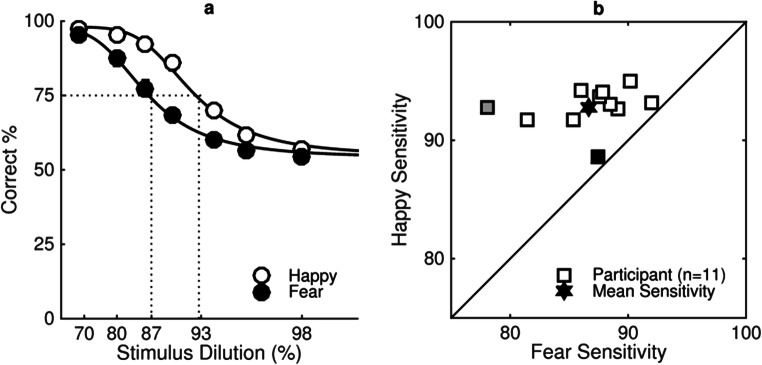
**a** Group sensitivity measure to happy (open symbols) and fearful (filled symbols) expressions. The percentage of trials participants (*n* = 11) correctly identified the expressive face as being more expressive than the neutral face is plotted as a function of stimulus dilution (%). **b** Individual sensitivity measures to happy (*y*-axis) and fearful (*x*-axis) expressions. Each square represents an individual participant’s data, and mean sensitivity is represented by the filled star. The grey square indicates the individual whose results are explored in more detail in Fig. 5. The black square indicates an individual for whom there is no statistical difference between sensitivity to happy and fear. Stimuli were presented for 200 ms, and participants sat 50 cm from the screen such that images subtended 19° × 27° of visual angle

Figure 3 shows a group sensitivity measure (see Fig. 3a) and individual sensitivity measures (Fig. 3b) to happy and fearful expressions. At the group level there is greater sensitivity to happy than to fearful facial expressions. This is evident by a rightwards shift in the psychometric function representing performance with happy expressions (open symbols) compared with the function representing performance with fearful expressions (filled symbols) in Fig. 3a. At the individual level, there is greater sensitivity to happy than to fearful expressions (*n* = 11). This is evident in Fig. 3b, in which all of the data points lie above the diagonal.

Figure 3a plots a Logistic function fit to *estimates* of percent correct (±*SEM*) as a function of stimulus dilution for happy (open symbols) and fearful (filled symbols) expressions in a temporal two-interval forced-choice task. Performance decreases from accurate (100%) to chance (50%) as stimulus dilution increases for both happy and fearful expressions. The fitted function representing estimates for happy expressions is shifted to the right of the function representing estimates for fearful expressions, indicating that participants are more sensitive to happy expressions.

Sensitivity is defined as measurement of the proportion of neutral facial expression in a stimulus required for participants to discriminate the emotional expression on 75% of presentations. Significantly more dilution is required for performance to reduce to 75% correct for happy expressions (*M* = 92, *SE* = .57) than for fearful expressions (*M* = 86, *SE* = 1.23), *t*(10) = 5.06, *p* < .0001, *r* = .85, as is illustrated by the dotted lines in Fig. 3a. This increase in dilution required for performance to reduce to 75% for happy expressions is described as greater sensitivity to happy than to fearful facial expressions.

**Fig. 4:**
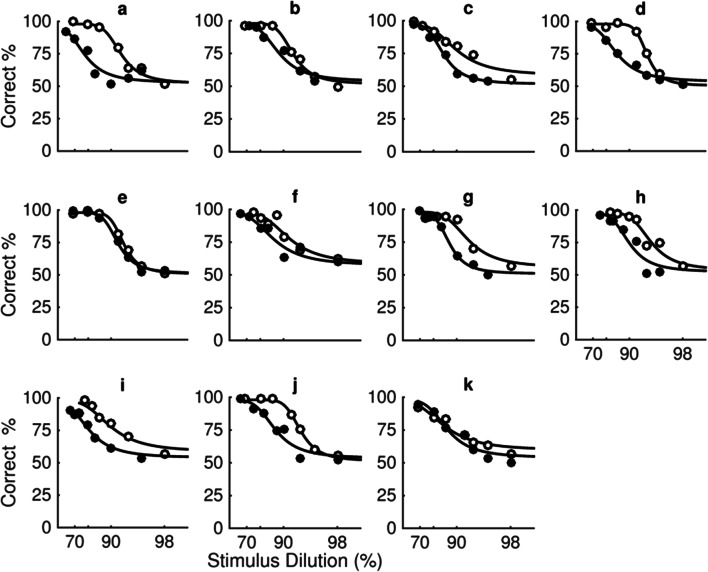
**a–k** shows the percentage of trials individual participants correctly identified the test stimulus as the most expressive plotted as a function of stimulus dilution (%) for happy (open symbols) and fearful (filled symbols) expressions averaged across all six unique faces. Fig. 4a and k show the psychometric functions indicated by the grey and black symbols of Fig. 3b, respectively. All other details as in Fig. 3

The estimates in Fig. 3a are derived using the parameters from separate psychometric functions fit using a Logistic function with the Palamedes toolbox (Prins & Kingdom, 2018) for each individual participant (see Fig. 4). In order to average data across all participants, estimates of percentage correct for a fixed set of seven dilutions (68%, 80%, 86%, 90%, 94%, 96%, 98%) were used.

**Fig. 5:**
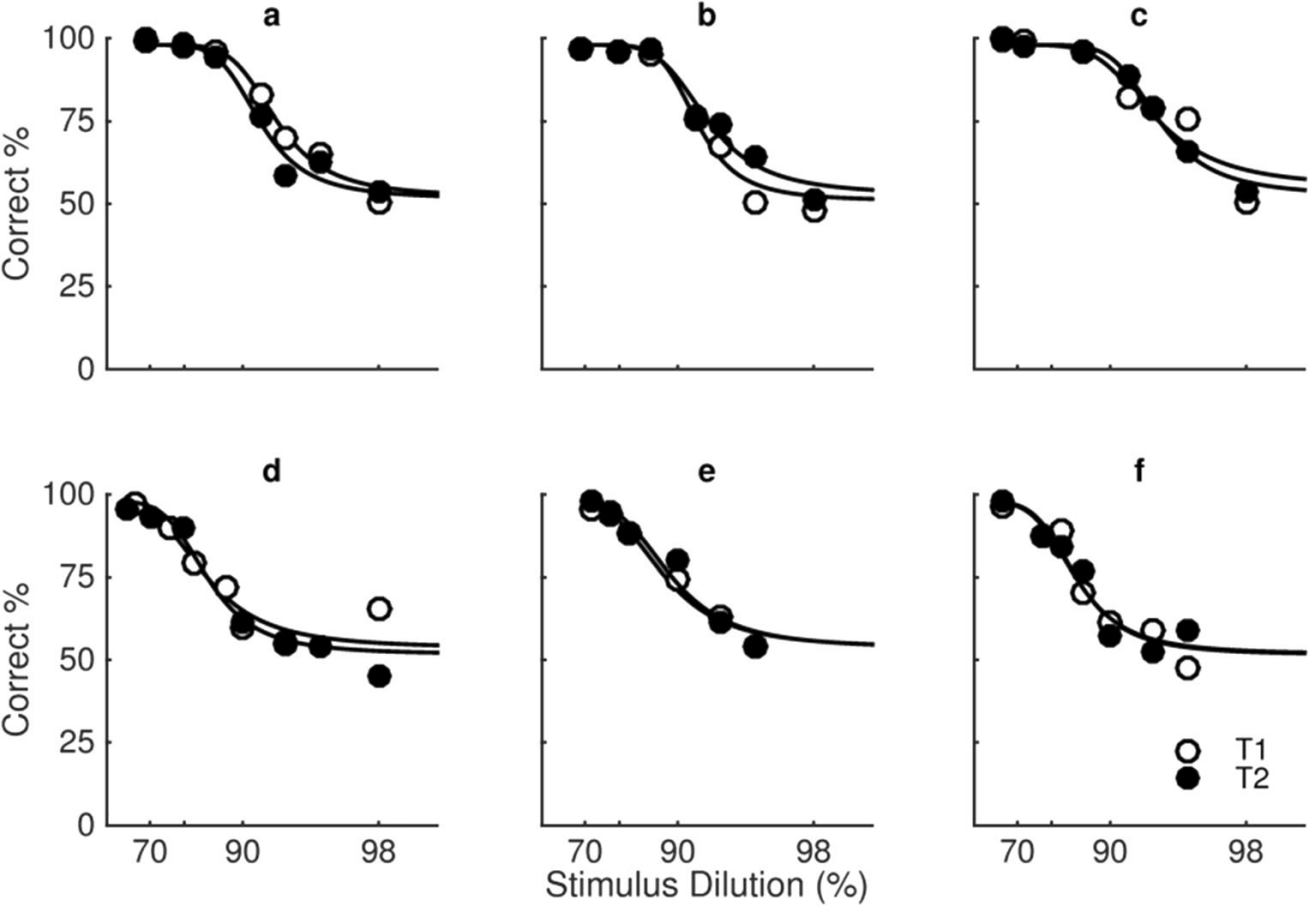
**a–f** shows the percentage of trials three individual participants (columns) correctly identified the test stimulus as the most expressive plotted as a function of stimulus dilution (%) measured at two different testing sessions (T1, open symbols and T2, filled symbols) for happy (top row) and fearful (bottom row) expressions averaged across all six unique faces. All other details as in Fig. 3

Figure 3b plots sensitivity to happy (*y*-axis) and fearful (*x*-axis) expressions estimated using psychometric functions fit to the performance of individual participants (*n* = 11) in the same temporal two-interval forced-choice task as the averaged data represented in Fig. 3a (see Fig. 4 for psychometric functions of each participant). Sensitivity is defined as measurement of the proportion of neutral facial expression in a stimulus required for participants to discriminate the emotional expression in 75% of presentations. The square data points represent sensitivity of individual participants to happy and fearful expressions. Each data point lies above the diagonal showing greater sensitivity to happy expressions than to fearful expressions for each individual participant. The grey square represents the individual whose results are explored in more detail in Fig. 5, whereas the filled star represents mean sensitivity averaged across all participants.

Figure 4 plots percentage correct as a function of stimulus dilution for happy (open symbols) and fearful (filled symbols) expressions for individual participants in the same temporal two-interval forced-choice task represented in Fig. 3. Performance decreases from accurate (100%) to chance (50%) as stimulus dilution increases for both happy and fearful expressions. The fitted functions representing performance for happy expressions are shifted to the right of the function representing fearful expressions. This indicates that participants are more sensitive to happy compared with fearful expressions averaged across unique faces.

A model comparison using the likelihood ratio test of the Palamedes toolbox for MATLAB was used to test the differences between functions fit to performance with happy and fear expressions for each individual participant. For 10 of the 11 participants, the differences in the fitted functions between the happy and fear expressions were real, with *p* < .0001 for seven participants and *p* < .01 for three. For one participant (shown in Fig. 4k and represented by the black square in Fig. 3b), there was no real difference in fitted functions for happy and fear (*p* = .31).

Figure 5 plots percentage correct as a function of stimulus dilution measured on two different testing sessions (open and filled symbols) for happy (top row) and fearful (bottom row) expressions for three individual participants (columns). A model comparison using the likelihood ratio test of the Palamedes toolbox for MATLAB was used to test the differences between functions fit to performance measured at testing Sessions 1 and 2 for each individual participant and each expression. There are no differences in the fitted functions between the testing sessions for any participant or expression (*p* > .10 in all cases).

### Experiment 2: Sensitivity to happy and fearful expressions at the unique face level

**Fig. 6:**
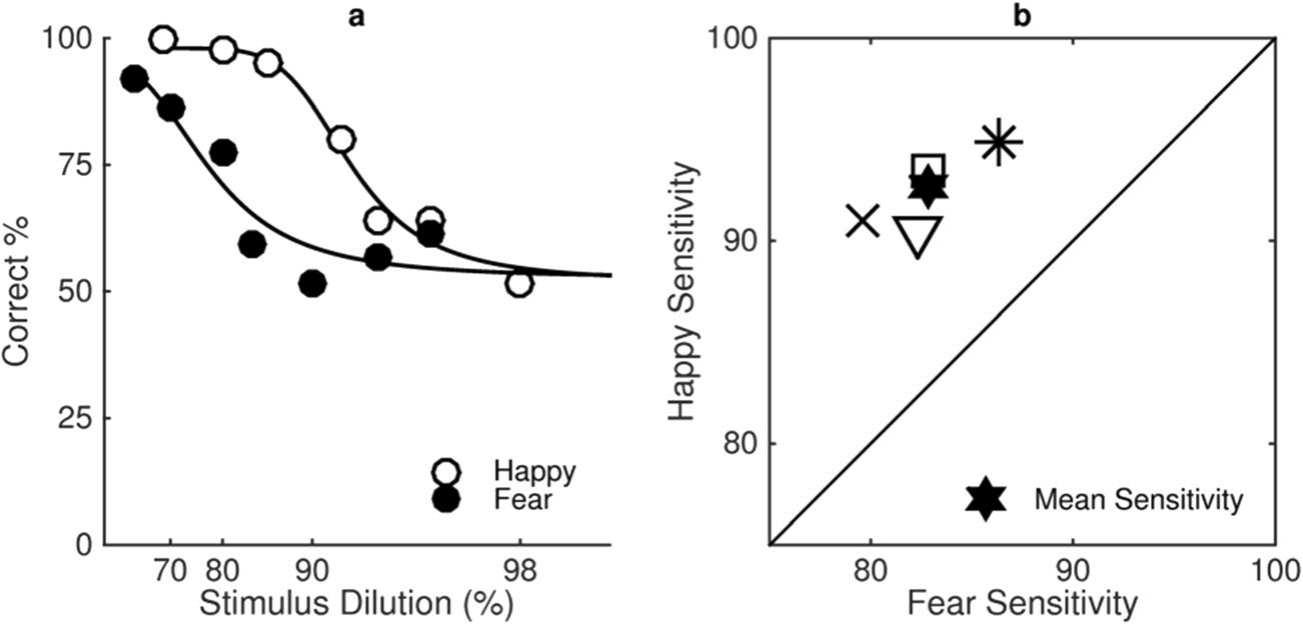
**a** shows the percentage of trials a single participant (*n* = 1) correctly identified the test stimulus as the most expressive plotted as a function of stimulus dilution (%) for happy (open symbols) and fearful (filled symbols) expressions averaged across all six unique faces. **b** shows sensitivity to happy (*y*-axis) and fearful (*x*-axis) expressions for each unique face (two female and two male faces) and mean sensitivity (filled star). It was not possible to fit a function to fearful expressions for FF2 and MF2 therefore their data points are missing. All other details as in Fig. 3

Individual participants are more sensitive to happy than to fearful facial expressions, and this is somewhat independent of unique face stimuli. Figure 6 represents performance of the individual identified by the grey square in Fig. 3b and is identical to the data in Fig. 4a. Greater sensitivity to happy expressions is evident by a rightwards shift in the psychometric function representing performance with happy compared with fearful expressions (see Fig. 6a). Greater sensitivity to happy than to fearful expressions is also evident by the data points representing unique face stimuli lying above the diagonal in Fig. 6b.

Figure 6a plots percentage correct as a function of stimulus dilution for happy (open symbols) and fearful (filled symbols) expressions in a temporal two-interval forced-choice task for one participant (same task as grey square in Fig. 3b and Fig. 4a). Performance decreases from accurate (100%) to chance (50%) as stimulus dilution increases for both happy and fearful expressions. The fitted function representing performance for happy expressions is shifted to the right of the function representing fearful expressions. This indicates that the participant is more sensitive to happy compared with fearful expressions averaged across unique faces.

**Fig. 7:**
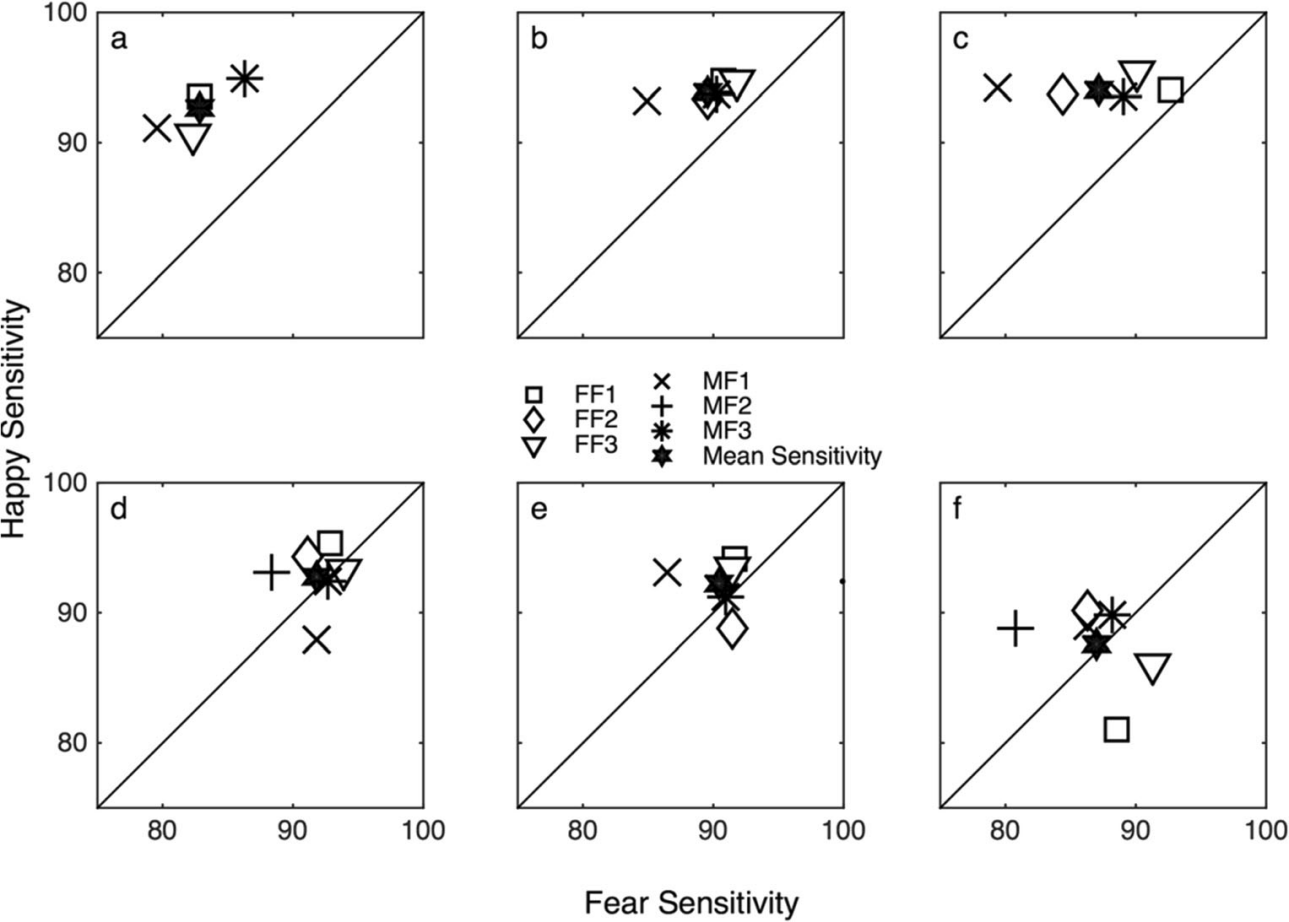
Sensitivity to happy (*y*-axis) and fearful (*x*-axis) expressions for six individual participants (a–f) for each unique face (three female [FF] and three male [MF] faces; see Fig. 2). Mean sensitivity is represented by a filled star. Figure 6b is replotted here as in Fig. 7a. All other details as in Fig. 3

Figure 6b plots sensitivity to happy (*y*-axis) and fearful (*x*-axis) expressions for each unique face (symbols). Sensitivity is estimated using psychometric functions fit to performance corresponding to each unique face. All of the data points lie above the diagonal for each unique face showing greater sensitivity to happy compared with fearful expressions. There is variability in the discriminability of unique faces evident by the spread in the data points in Fig. 6b. Figure 7 explores whether there is variability in the discriminability of unique faces for other participants.

While individual participants are more sensitive to happy than to fearful facial expressions, there is variability in the discriminability of unique faces. Figure 7 plots sensitivity to happy (*y*-axis) and fearful (*x*-axis) expressions for each unique face (different symbols) for six individual participants (Fig. 7a–f). Mean sensitivity is represented by the filled star, and unique faces are represented by symbols (see Fig. 2 for examples of the unique faces). Where it was not possible to fit a function to data (performance with fearful faces in Fig. 7a, b, c, and e), sensitivity to those unique faces is not plotted. In such cases, performance with the fear expressions was not high enough to generate a full psychometric function at the low stimulus dilutions presented. In all cases, it was possible to fit a function to each unique happy face.

There is greater sensitivity to happy than to fearful faces for all participants averaged across all unique faces. This is evident by mean sensitivity (across unique face; filled star) lying above the diagonal. However, there is variability in the magnitude of the difference in sensitivity to happy and fearful expressions. Mean sensitivity for two participants is very close to the diagonal line (Fig. 7d and f). This indicates that there is little difference in discriminability between happy and fearful expressions for these participants.

Figure 6 also shows variability in sensitivity for unique faces, indicating that the unique faces are not perceptually equivalent. Some participants show greater sensitivity to all unique happy face stimuli (Fig. 7a–c), while other participants show greater sensitivity to some unique fearful face stimuli (Fig. 7d–f). This is evident by the data points lying above and below the diagonal line, respectively. This variability suggests that unique faces are not perceptually equivalent. Individual differences in ability to discriminate different faces do not follow an obviously consistent pattern indicating differences in perception rather than properties of the stimuli themselves. Experiment 3 tests whether discriminability of happy and fearful expressions is influenced by stimulus duration.

### Experiment 3: Effect of stimulus duration on sensitivity to happy and fearful expressions

**Fig. 8:**
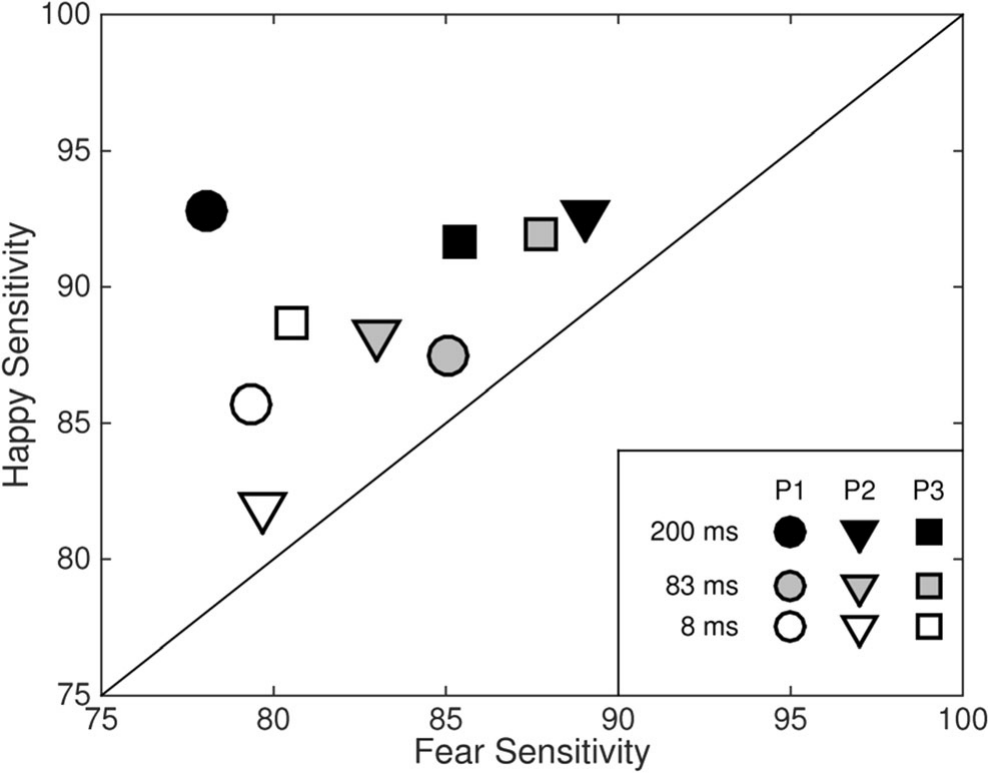
Sensitivity to happy (*y*-axis) and fearful (*x*-axis) expressions for three individuals (P1–P3; symbol) for three different durations (8, 83, and 200 ms; fill). Participants sat 50 cm from the display, and images subtended 19° × 27° of visual angle

Individual participants are more sensitive to happy than to fearful facial expressions, and this is independent of stimulus duration. Figure 8 plots sensitivity to happy (*y*-axis) and fearful (*x*-axis) expressions for three individual participants (symbol) at three different stimulus durations (8 ms, 83 ms, and 200 ms; fill shade).

There is greater sensitivity to happy expressions than to fearful expressions for all participants at all stimulus durations. This is evident by all of the data points lying above the diagonal (see Fig. 8). Sensitivity tends to be greater at longer stimulus durations and lesser at shorter stimulus durations (compare filled and open symbols).

A model comparison using the likelihood ratio test (Prins & Kingdom, 2018) of the Palamedes toolbox (Prins & Kingdom, 2018) for MATLAB was used to test the differences between functions fit to performance with happy and fear expressions for each individual participant. For all three participants, the differences in the fitted functions between the happy and fear conditions at each stimulus duration were real, with *p* < .0001 in eight cases and *p* < .05 in one. Experiment 4 tests whether image size affects the discriminability of happy and fearful expressions.

### Experiment 4: Effect of image size on sensitivity to happy and fearful expressions

**Fig. 9:**
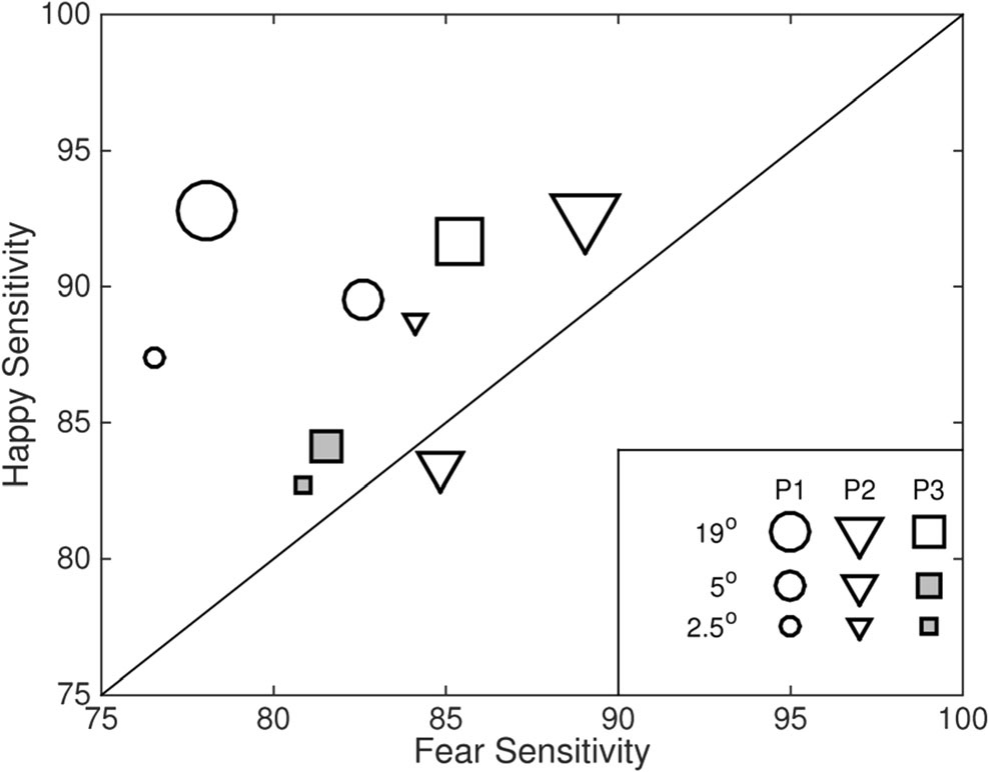
Sensitivity to happy (*y*-axis) and fearful (*x*-axis) expressions for three individuals (P1–P3; symbol) for three different image sizes (19° × 27°, 5° × 7° and 2.5° × 3.5° of visual angle; size). Stimuli were presented for 200 ms

Individual participants are more sensitive to happy than to fearful facial expressions, and this is largely independent of image size. Figure 9 plots sensitivity to happy (*y*-axis) and fearful (*x*-axis) expressions for three individual participants (symbol) with three different image sizes (19° × 27°, 5° × 7°, and 2.5° × 3.5° of visual angle; symbol size). Changes in image size were achieved by changing the distance that participants sat from the screen (50 cm, 200 cm, and 400 cm, respectively).

There is greater sensitivity to happy expressions than to fearful expressions for most participants and most image sizes. This is evident by most data points lying above the diagonal. For one participant (triangles), there is greater sensitivity to fear expressions with images subtending 5° × 7° of visual angle. This is evident by the data point lying below the diagonal.

A model comparison using the likelihood ratio test of the Palamedes toolbox for MATLAB was used to test the differences between functions fit to performance with happy and fear expressions for individual participants. For two out of three participants, the differences in the fitted functions between the happy and fear conditions at each stimulus duration were real. In most of these cases, sensitivity to happy was greater than fear, *p* < .0001 (open symbols); however, Participant 2 (P2) was more sensitive to fear for faces that subtended 5° × 7° of visual angle (*p* < .01). For Participant 3 (P3), there was no real difference in fitted functions for happy and fear for images subtending 5° × 7° and 2.5° ^×^ 3.5° of visual angle (*p* = .05 and *p* = .18, respectively; grey symbols). Experiment 5 tests whether the morphing software used to generate stimuli affects the discriminability of happy and fearful expressions.

### Experiment 5: Effect of morphing software on sensitivity to happy and fearful expressions

Individual participants are more sensitive to happy than to fearful expressions, and this is independent of the morphing software used to generate stimuli. Nine new participants took part in a replication of Experiment 1 with stimuli generated using a different morphing software (Psychomorph; see Methods for full details).

Mean (*SE*) sensitivity to stimuli generated using Psychomorph is 93% (0.91) for happy and 86% (1.21) for fearful expressions. This is comparable to mean (*SE*) sensitivity to stimuli generated using Norrkross MorphX (Experiment 1); 92% (.57) for happy and 86% (1.23) for fearful expressions. A paired-samples *t* test shows that participants are more sensitive to happy than to fearful expressions when stimuli are generated using Psychomorph, *t*(8) = 5.96, *p* < .0001, *r* = .9, as they were with stimuli generated using Norrkross MorphX (see Experiment 1). There are no significant differences between sensitivity to happy (or fearful) stimuli generated using Norrkross MorphX and Psychomorph. This suggests that greater sensitivity to happy than to fearful expressions is reliable and independent of the morphing software and process used to generate stimuli.

## Discussion

This paper describes a method to measure an individual’s sensitivity to facial expressions conveying different emotions (happy and fear) as well as a method to test whether these differences are statistically significant for the individual. Individual participants are more sensitive to happy than to fearful facial expressions, and this is independent of the testing session, stimulus duration and morphing software used to generate the images. This demonstrates that greater sensitivity to happy than to fearful expressions is a finding which is measurable at the level of the individual.

The approach described in this paper is a sensitive measure of the ability of participants to discriminate stimuli with different facial expressions. The results show that individual participants are more sensitive to happy expressions than to fear expressions, and that these differences are statistically significant using the model-comparison approach. Statistical differences are also evident at the group level with greater sensitivity to happy than to fearful expressions with relatively small sample sizes (*n* = 11 and *n* = 9) and large effect sizes (*r* > .85). These findings are also consistent with research showing an increase in accuracy and a decrease in reaction times in response to happy expressions when analyzed at a group level (Calder, Keane, et al., 2000a; Calvo & Lundqvist, 2008; Calvo & Nummenmaa, 2009; Matsumoto & Hwang, 2014; Palermo & Coltheart, 2004; Recio et al., 2013).

While individual participants are more sensitive to happy than to fearful facial expressions, this paper shows that if performance is analyzed at the level of unique face stimuli (e.g., specific female and male faces), there are differences. Some participants show greater sensitivity to all unique happy face stimuli, while other participants show greater sensitivity to some unique fearful face stimuli. This suggests that unique face stimuli are not perceptually equivalent.

It was not possible to fit psychometric functions to some unique faces expressing fear. The seven levels of stimulus dilution were constant across unique faces, as their presentation was randomly interleaved, rather than in blocks. The weakest stimulus dilution presented was still too strong for performance to be greater than ~75% correct for some unique faces. This challenge is similar to that faced by Marneweck et al. (2013), when they found that standardizing the levels of stimulus intensity prevented psychometric functions being fit to all participants data. To overcome this, unique face stimuli could be presented in blocks using different stimulus dilutions to characterize the full relationship with performance.

Individual differences in ability to discriminate unique face stimuli do not follow an obviously consistent pattern and suggest a difference in perception rather than a property of the stimuli. Understanding these individual differences requires further research. To overcome any potential issues with unique faces not being equivalent, future research should consider the use of prototypical face stimuli generated using the average of a number of unique faces (Tiddeman et al., 2001).

This paper used validated images from the Radboud Face Database. Despite the average intensity ratings of the set of happy and fearful faces used in this paper being the same (4.25), it is clear that individuals are more sensitive to happy than to fearful expressions. There are two possible explanations for this: (i) The visual signals corresponding to happy expressions are stronger or more salient and therefore easier to detect than those of fearful expressions, and/or (ii) individuals are more sensitive to the [positive] affect conveyed by happy expressions than that conveyed by fearful expressions.

This paper shows that individuals are more sensitive to happy than to fearful expressions even when stimuli are presented for 8-ms duration. This suggests that the visual signals in the stimuli play a significant role in performance on this task compared with the role of the affect conveyed by the stimuli—which presumably would require additional processing time. Calvo and Nummenmaa (2016) recently summarized the literature investigating the role of perceptual and affective processing in facial expression recognition. In line with data presented in this paper, they suggest that when measuring behavioural performance on an emotion recognition task, existing methodological approaches typically rely more heavily on the visual signals in the stimuli than the affect conveyed. Calibrating stimuli to individual participants by generating stimulus sets that are perceptually equivalent could overcome this bias and allow dissociation of the perceptual salience of the visual signals from the affect conveyed.

This paper measures the relationship between accuracy and stimulus dilution for an individual on a discrimination task with stimuli conveying two different emotions. Knowing this relationship makes it possible to define stimuli that are behaviourally equivalent for individual participants (e.g., stimulus dilution required to perform at 75% correct for each stimulus condition for each participant) yet belong to distinct affective categories (e.g., happy or fear). Such stimuli could be described as being *perceptually equivalent* and the relationship defined by this method can be used to generate a personalized stimulus set tailored to each participant. Each set would comprise images with variable stimulus dilutions, each of which meet a defined behavioural criterion. Using this personalized stimulus set in a different behavioural paradigm (e.g., categorical emotion recognition task) would reduce the contribution of the salience of the visual signals (perceptual processing) and amplify the relative contribution of affective processing. Calibrating stimuli in this way could allow dissociation of the perceptual salience of the visual signals from the affect conveyed so that one could be measured independent of the other. Comparing performance with and without perceptually equivalent stimuli could provide greater insight into the relative contribution of perceptual and affective processing in facial emotion recognition tasks.

The relative importance of the visual signals in emotion recognition may also be demonstrated by the finding that varying the image size, by changing the viewing distance from the screen, affects sensitivity to emotions for two of the three participants tested. At smaller images sizes these participants were more likely to show no difference in sensitivity to happy and fear expressions (Participant 3) or show increased sensitivity to fear expressions (5° image; Participant 2). This could be related to the role of the spatial frequency information in the images carrying the affect and how this is altered as image size decreases. Zeev-Wolf and Rassovsky (2020) showed that participants are more accurate and respond more quickly when happy and fear expressions are filtered to include low-frequency information. However, this is not the case for people with schizophrenia, who find it more difficult to recognize emotion from images with low-spatial-frequency information compared with broad or high-spatial-frequency information (Jahshan, Wolf, Karbi, Shamir, & Rassovsky, 2017). In view of this, care should be taken to control for image size, in particular if using the methodology outside of the lab and comparing performance between control and clinical populations.

One disadvantage of the approach described here is that it can be quite time-consuming to measure the individual’s sensitivity to each emotion. As the task is self-paced and participants are encouraged to take short rest breaks between trials if needed, it can take between 20 and 30 minutes per emotion. If the main goal of the research is to identify sensitivity to specific emotions (i.e., identify stimulus dilution at a given behavioural criterion) for a group of participants, then an adaptive version of the task may be more efficient. Using an adaptive method, the computer would select the stimulus dilution to be presented on the next trial based on the performance of the participant on previous trial(s) until a given behavioural criterion is reached (Alcalá-Quintana & García-Pérez, 2007).

This paper describes a robust method for measuring an individual’s sensitivity to different facial expressions. Future research in the lab is concerned with (i) measuring the sensitivity of *individuals* (from distinct clinical populations) to different intensities of emotions with a view to developing a tool to support clinical diagnosis and/or treatment, and (ii) developing personalised stimulus sets tailored to individual participants to explore the relative contribution of *perceptual processing* and *affective processing* in facial expression recognition.
